# Esthetic Composition of Smile in Individuals with Cleft Lip, Alveolus, and Palate: Visibility of the Periodontium and the Esthetics of Smile

**DOI:** 10.1155/2012/563734

**Published:** 2012-11-21

**Authors:** Luis Augusto Esper, Michyele Cristhiane Sbrana, Mércia Jussara da Silva Cunha, Guilherme Santos Moreira, Ana Lúcia Pompéia Fraga de Almeida

**Affiliations:** ^1^Oral Rehabilitation, Bauru School of Dentistry, University of São Paulo, 17012-901 Bauru, Brazil; ^2^Setor de Periodontia do Hospital de Reabilitação de Anomalias Craniofaciais da Universidade de São Paulo, Rua Silvio Marchione, 3-20 Vila Universitária, 17012900 Bauru, SP, Brazil; ^3^Department of Prosthodontics at Bauru School of Dentistry, University of São Paulo, 17012-901 Bauru, SP, Brazil

## Abstract

*Objective*. To evaluate characteristics of smile related to visibility in individuals with cleft lip, alveolus, and palate. *Design*. Cross-sectional. *Setting*. HRAC/USP, Brazil. *Patients*. Individuals with repaired complete unilateral cleft lip and palate (*n* = 45), aged 15–30 years. *Interventions*. Frontal facial photographs were obtained in natural and forced smiles (*n* = 135). Six specialists in periodontics evaluated the photographs as to the smile line, thickness, and curve of the upper lip. *Main Outcome Measures*. The cleft area was compared with the contralateral region. Results were expressed as percentages and means. The findings were compared between groups of periodontists. *Results*. Statistically significant relationship was observed in the smile line between examiners and between natural and forced smiles, regardless of the association with the cleft side. The lip was thicker at rest and thinner in the forced smile, as also evaluated by the group not experienced with cleft care. The curve of the upper lip in natural and forced smiles was considered as close to straight by both groups, regardless of the cleft. *Conclusion*. The smile in individuals with clefts was regarded as average for both cleft and noncleft sides. The thickness was characterized as average to thin, being thinner in forced smile and when analyzed by the group not experienced with cleft care. In the average, the curve of the upper lip was considered as straight. The present study elucidates some characteristics related to the smile in individuals with repaired unilateral cleft lip, alveolus, and palate.

## 1. Introduction and Literature Review

The facial appearance is a key element in the psychosocial development of individuals with cleft lip, alveolus, and palate, who usually present characteristics of social introversion [1, 2]. Their rehabilitation should be conducted with a view to enhance the functional and esthetics characteristics and consequently the quality of life.

The smile is one of the most important facial expressions and should be carefully analyzed as a whole before the accomplishment of dental treatment, aiming to establish the harmony between the teeth and adjacent soft tissues, achieving an esthetic and pleasant smile [3]. Evaluation of the face should be conducted together with the intraoral examination for the establishment of treatment goals that may meet the patient's expectations and reestablish the most esthetic conditions as possible [4].

The esthetic analysis of smile by the visual perception should consider it as a unique composition, in which some elements act in combination to make the smile harmonious and pleasant for the observer. Significant disruptions in the composition deviate the attention to the undesired element; alterations in the gingival component, lip shape, and filling of the gingival papillae call the attention due to their distinguished role in the composition of smile [5].

Concerning the position of the upper lip, the smile may be classified as high, average, and low [6], which is very important in the indication of periodontal plastic surgeries and restorative procedures performed in esthetic areas. The high smile reveals the entire cervicoincisal length of maxillary anterior teeth and the adjacent gingiva; in the average smile, 75% to 100% of the maxillary anterior teeth and the interproximal gingiva are observed; in the low smile, less than 75% of the anterior teeth are visible. Higher smiles usually expose the gingival margin and restorations in esthetic areas, and any imbalance may affect the esthetics [7].

Few studies have analyzed the teeth and visibility of the soft tissue periodontium (gingival tissue) during smile. Liébert et al. [8] suggested a classification of the smile line (both in the normal and reduced periodontium) considering the visibility of the periodontium and gingival embrasures during natural and forced smiles.

The thickness of lips should also be observed, since individuals with thinner upper lip usually present greater exposure of teeth and higher smile compared to individuals with thicker lips.

Analysis of the symmetry of smile is very important, yet the curve of the upper lip should also be considered, based on the position of the mouth angle in relation to the center of the lower border of the upper lip, according to the following three categories: ascendant, descendant, or straight, being that the ascendant and straight patterns are considered more esthetic [9].

The literature on the aspect of the lip in individuals with unilateral cleft lip and palate mentions the aspect of scar contraction and muscle misalignment, causing esthetic and functional damages to these individuals after primary cheiloplasty surgeries [10]. Some studies suggest that the upper lip in these individuals differs from the normal upper lip because it presents lower elasticity and shortened height [11].

Some authors consider the average smile as more frequent in the population (ranging from 56% to 68.94%), followed by the low smile (15% to 20.48%), and high smile (10.48% to 29%). Therefore, in most cases, only the interproximal papillae are visible during smile [6, 12].

The adequate contour of the gingival tissue following the regular concave shape and filling of the interdental spaces by the interproximal papillae are extremely important in the analysis of the gingival component of smile and its esthetic aspect as a whole [13]. A harmonious gingival contour is observed in the composition of smile in which the teeth present adequate relationship with the gingival tissue, since changes in the gingival contour may disturb the harmony of smile and impair the overall facial esthetics of the individual.

This study evaluated the characteristics of smile directly related to the visibility of the periodontium during natural and forced smiles in individuals with unilateral cleft lip, alveolus, and palate, and the possible influence from the cleft on the smile, comparing the evaluation of specialists in periodontics experienced or not with cleft care.

## 2. Material and Methods

The sample was composed of 45 Caucasoid individuals with unilateral cleft lip, alveolus, and palate aged 15 to 30 years, of both genders, repaired as to the dental treatment, and who had received definitive discharge from the Plastic Surgery sector.

Individuals with syndromes, presenting facial paralysis or using anticonvulsant drugs upon selection were excluded from the sample.

Three photographs were obtained from the face of each individual in the selected sample, in frontal view at the following positions: at rest, during natural, and forced smiles, adding up to 135 photographs (Nikon, model Coolpix 8700 ZOOM NIKKOR ED 8.9–71.2 mm 1 : 2.8–4.2 35–280 mm, 8.0 megapixels), for evaluation of the smile line, curve and thickness of the upper lip on each smile. In order to establish the adequate standardization, the photographs were obtained in a photo studio in natural head positioning of the individual on the cephalostat, with the camera placed on a rigid support located at a distance of 97 cm from the cephalostat [14].

**Figure 1:**
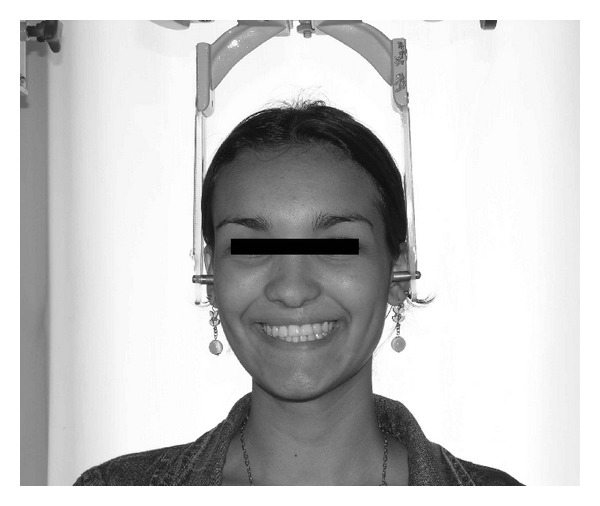
Photograph of patient in position, with the olives of the cephalostat placed in the cartilaginous tragus, during natural smile.

During the achievement of photographs, the individuals were maintained in straight position, with the feet 10 cm apart, looking at the camera ahead them with the infraorbital plane parallel to the ground. In all photographs, the olives of the cephalostat were introduced in the cartilaginous tragus keeping light contact with the skin, to prevent the individual from raising the head and neck (Figure 1).

The photographs were analyzed by two groups of examiners, composed of 6 dentists specialists in periodontics, one composed of 3 periodontists experienced with cleft care (group 1), and another composed of 3 periodontists not experienced with cleft care (group 2). The 135 photographs were individually analyzed by the examiners, at different orders of the individuals, and each side of the smile was evaluated in relation to the midline without previous knowledge on the cleft side.

Concerning the smile line, the examiners were asked to classify the smile into one of the categories proposed by Liébert et al. [8].

**Figure 2:**
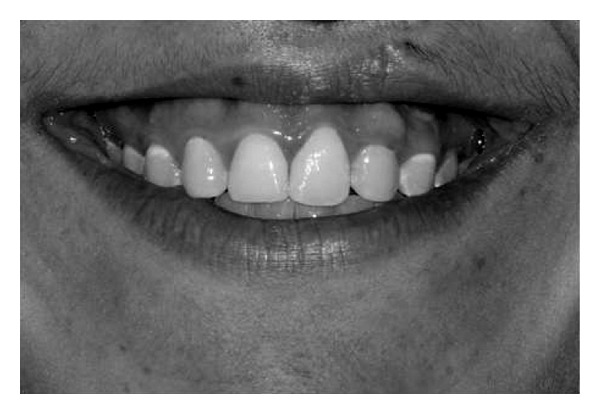
Very high smile.

**Figure 3:**
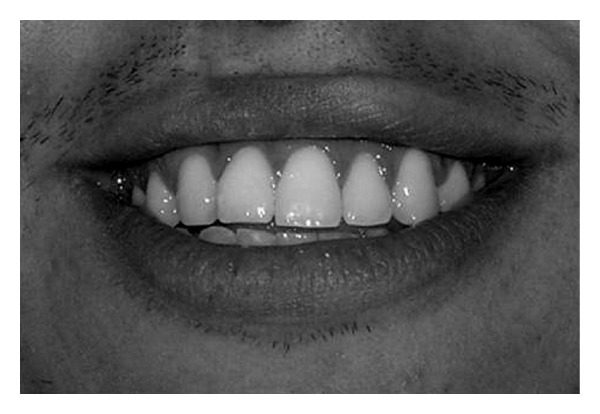
High smile.

**Figure 4:**
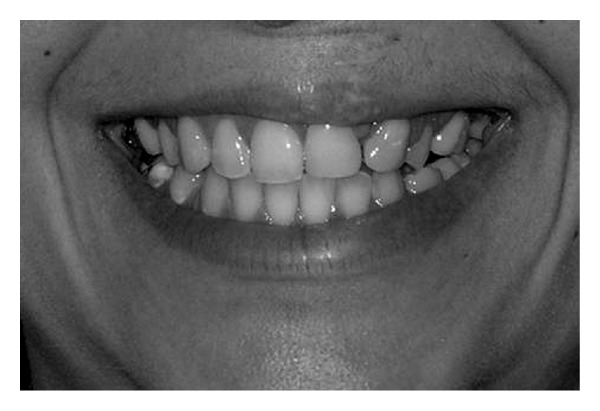
Average smile.

**Figure 5:**
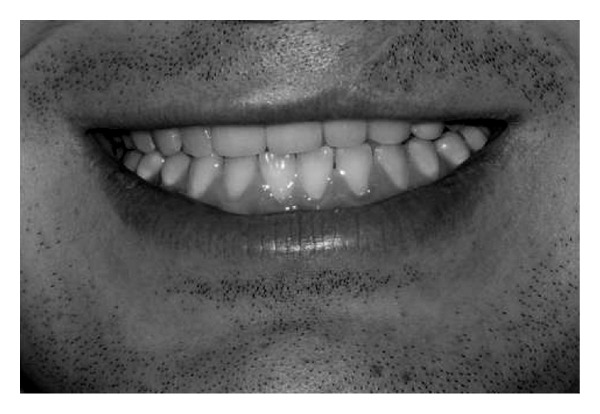
Low smile.

- Class 1—Very high smile line: more than 2 mm of the marginal gingiva visible or more than 2 mm apical to the cementoenamel junction visible for the reduced but healthy periodontium (Figure 2).
- Class 2—High smile line: between 0 and 2 mm of marginal gingiva visible or between 0 and 2 mm apical to the cementoenamel junction visible for the reduced but healthy periodontium (Figure 3).
- Class 3—Average smile line: only gingival embrasures visible (Figure 4).
- Class 4—Low smile line: gingival embrasures and cementoenamel junction not visible (Figure 5).

**Figure 6:**
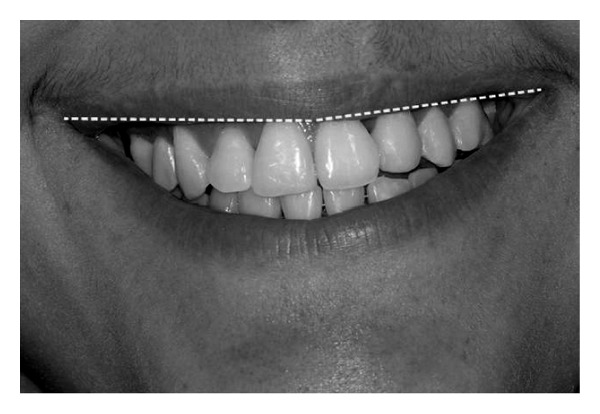
Straight curve of the upper lip at the patient's right side and slightly ascendant curve of the upper lip at the patient's left side.

**Figure 7:**
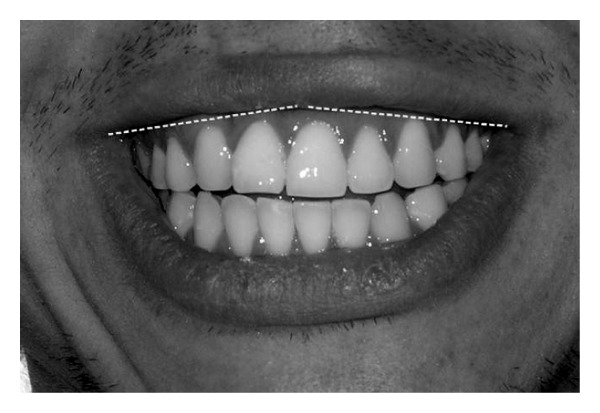
Descendant curve of the lip at both sides.

All examiners classified the photographs during smile and at rest according to a score ranging from 1 to 9, as follows: 1, 2, and 3 represented unpleasant esthetics; 4, 5, and 6 indicated acceptable esthetics; 7, 8, and 9 revealed pleasant esthetics [15]. Concerning the thickness, the examiners were asked to classify the lip on each photograph as: thick (score 2), intermediate or average (score 1), or thin (score 0) (at rest and during each smile). The examiners also scored the curve of the upper lip as ascendant (score 1), straight (score 2), or descendant (score 3) during natural or forced smiles, characterized by the relationship between the lower border of the upper lip and the mouth angle (Figures 6 and 7).

The results were statistically evaluated by analysis of variance (ANOVA) for repeated measurements, considering an unstructured covariance matrix, at a significance level of 5%.

This study was approved by the Institutional Review Board of HRAC/USP.

## 3. Results

### 3.1. Smile Line

Evaluation of the classification of smile during natural smile at the noncleft side revealed predominance of average and low smile lines, accounting for 44.44% and 41.48% of cases, respectively, while the very high smile line was the least prevalent, being observed in only 2.96% of cases. At the cleft side, 55.19% of cases presented low smile line and 2.59% very high smile line (Table 1).

During forced smile, at the noncleft side, the average smile was the most prevalent, accounting for 40% of cases, and the very high smile line was the least prevalent (10.74%). At the cleft side, 35.19% were characterized as average smile line and 8.89% as very high smile line (Table 2).

Comparison of the mean scores in the evaluations by both groups of examiners, during natural smile, group 1 indicated the average smile at both cleft (3.19) and noncleft sides (3.33). Group 2 also classified the smile line as average at the cleft (3.31) and noncleft sides (3.46). During forced smile, the examiners in group considered the smile as close to the average (2.6) at the cleft and noncleft sides (2.75). Group 2 also evaluated the smile as close to the average at the cleft (2.74) and noncleft sides (2.90) (Table 3).

There was statistically significant relationship in the smile line between the groups of examiners and between the natural and forced smiles, regardless of the association with the cleft side. The scores were higher during natural smile compared to the forced smile, and group 2 assigned the highest scores (*P* < .0001) (Table 3).

Concerning the evaluation by gender, during natural smile, both groups scored the smile as close to the average and the female gender exhibited lower scores compared to the male gender, for both cleft and noncleft sides. However, there was no statistically significant difference between genders when evaluated by the two groups (*P* > .05) (Tables 4 and 5).

### 3.2. Thickness of Upper Lip

At rest, the thickness of the upper lip was close to the average at the cleft side when evaluated by groups 1 and 2. This was also observed at the noncleft side.

During natural and forced smiles, the lip was considered close to the average by group 1 and closer to thin by group 2, at both sides.

There were statistically significant associations between the type of smiles and the thickness of upper lip, as well as between the thickness and the groups of examiners, being the lip thicker at rest and thinner at forced smile, also when evaluated group 2 (Table 6).

### 3.3. Curve of the Upper Lip

The curve of the upper lip at natural and forced smiles, regardless of the cleft, was considered as close to straight by the two groups, yet statistically significant differences were found between groups (*P* < .0001). The score assigned to all cases was higher for group 1 compared to group 2, being closer to the score representing the straight smile concerning the curve of the upper lip (*P* < .050) (Table 7).

## 4. Discussion

Understanding the characteristics of a smile is extremely important to guide the dentist in the establishment of the principles of esthetics [9]. The smile line is one of the first characteristics that should be observed before any rehabilitative treatment in esthetic areas, since any disruption in the balance between the teeth, lips, and gingiva may become visible during speech or forced smile, impairing the esthetics [16, 17].

The characteristics of the lips are directly related to the smile, with influence of lip shape and volume on the quantity of teeth and gingival tissue exposed at rest, function, and smile [18]. Even though many individuals do not expose the gingival tissue during smile, those with a shortened upper lip or presenting lip hypermobility usually expose gingival tissue during the wide smile [19].

Concerning the smile line in the population, some authors state that the average smile is the most prevalent [6]. In the literature review on the principles to be considered in the esthetics of smile, the average smile line was also the most prevalent, followed by the high smile line (29%), being the low smile line the least prevalent (15%) [9, 12].

Even though the literature on the characteristics of smile in individuals without clefts is thorough, few studies have reported the characteristics of smile in individuals with clefts.

The findings of Liébert et al. [8] reported that, at the age of 21 to 35 years, the average smile line was more frequent during natural (50%) and forced smile (46.3%), and that a higher frequency of exposure of the periodontium was observed in the forced smile (93.3%) compared to the natural smile (64.9%) at this age range. In this study, there was predominance of low smile line at the cleft side and average smile line at the noncleft side during natural smile, suggesting greater visibility of the periodontium at the noncleft side, in which only the papillae are visible in the average.

Comparing the forced and natural smiles at both sides, there was greater exposure of teeth and gingival tissue, because the smile was closer to the average smile line. Classes 1 and 2 (that expose the gingival margin) at the noncleft side were increased from 14.07% to 42.89%, while at the cleft side the percentages were increased from 11.48% to 34.82%.

There are reports that the high smile line is more common among females at a ratio of 2 : 1, while the low smile line is predominant in males at a ratio of 2.5 : 1 [20]. In the present study, the females presented lower scores in both natural and forced smiles.

The assignment of higher scores by the group of specialists not experienced with cleft care may be related to the fact that they do not present detailed knowledge on the cleft condition and present a more critical view because they consider individuals without clefts as standard. We assume that individuals with clefts may present less lip mobility and consequently less exposure of teeth and gingival tissue, yet this analysis should be carefully performed.

The thickness of the upper lip was greater at rest, being decreased in the natural smile, and thinner in the forced smile. Even though the literature on individuals without clefts reports that individuals with thin upper lips tend to present high smile line and greater exposure of teeth, this relationship was not observed among individuals with clefts. A more critical evaluation may have been conducted by the examiners in group 2, who scored the thickness of the upper lip as thin in natural and forced smiles. The thickness of the lips may also be influenced by the plastic surgeries (cheiloplasty) to which these individuals are submitted [21] and did not determine a greater smile line in these individuals.

According to Dong et al. [9], the curve of the upper lip is considered as more esthetic when it is straight or slightly ascendant. In the present study, in most cases, the curve of the upper lip during smile was characterized as straight; in the comparison between evaluations, group 2 may have probably considered the curve as ascendant in more evaluations. Little clinical significance may be assigned to this difference between groups, since ascendant and straight curves are reported in the literature as being more esthetic compared to the descendant [9].

It should be highlighted that the evaluation of smile should be performed before the planning of oral rehabilitation, considering the characteristics of the lips that may interfere with the visibility of the periodontium and the esthetics of smile.

## 5. Conclusions

It may be concluded that the smile line in individuals with unilateral cleft lip, alveolus, and palate varied according to the type of smiles (natural or forced), being scored as average at the cleft and noncleft sides, with low prevalence of high and very high. During forced smile, both the cleft and non-cleft sides were scored as average, yet with an increase in the proportion of high and very high smile lines.

The thickness of the upper lip was considered from average to thin, being the lip thinner during forced smile. The curve of the upper lip was predominantly considered as straight.

Several characteristics should be evaluated in the dentolabial analysis, combined with the facial analysis for the determination of esthetics. The group of examiners not experienced with cleft care considered the characteristics of smile in a more critical manner, because they consider individuals without clefts as standard. The cleft side may play a role in the esthetics of smile, with lower visibility of the periodontium at this area, which is extremely important in the clinical practice when planning the oral rehabilitation of these individuals.

The esthetic principles are well established for individuals without clefts, yet there is lack of studies delineating these characteristics in individuals with clefts. This study meets these expectations and elucidates some characteristics related to the smile in individuals with repaired unilateral cleft lip, alveolus, and palate.

## Figures and Tables

**Table 1:** Height of smile.

Height of smile	Smile types							
	Natural (*n*) NC side	Natural (%) NC side	Natural (*n*) C side	Natural (%) C side	Forced (*n*) NC side	Forced (%) NC side	Forced (*n*) C side	Forced (%) C side
Very high (class 1)	8	2.96%	7	2.59%	29	10.74%	24	8.89%
High (class 2)	30	11.11%	24	8.89%	86	31.85%	70	25.93%
Average (class 3)	120	44.44%	90	33.33%	108	40.00%	95	35.19%
Low (class 4)	112	41.48%	149	55.19%	47	17.41%	81	30.00%

Total	270	100%	270	100%	270	100%	270	100%

**Table 2:** Men scores in the evaluation by both groups of examiners.

Effect	Estimate	Confidence interval lower limit	Confidence interval upper limit
Group 1-Group 2*	−0.1352	−0.2238	−0.04652
Natural–Forced*	0.5759	0.3420	0.8098

*statistically significant (*P* < .05).

**Table 3:** Estimate of mean differences in the scores of smiles (ANOVA).

Side	Smile type	Examiner group	Mean score	Standard deviation
Cleft side	Natural	Group 1	3.19	0.76
		Group 2	3.31	0.69
	Forced	Group 1	2.60	0.87
		Group 2	2.74	0.83

Non cleft side	Natural	Group 1	3.33	0.72
		Group 2	3.46	0.70
	Forced	Group 1	2.75	0.88
		Group 2	2.90	0.86

**Table 4:** Mean scores in the evaluations of group 1 according to gender.

Side	Smile type	Gender	Mean score	Standard deviation
Cleft side	Natural	Male	3.41	0.61
		Female	2.89	0.77
	Forced	Male	2.70	0.94
		Female	2.47	0.76

Non cleft side	Natural	Male	3.52	0.56
		Female	3.07	0.83
	Forced	Male	2.84	0.83
		Female	2.63	0.94

**Table 5:** Mean lip thickness.

Smile type	Side	Examiner group	Mean score	Standard deviation
Rest	Cleft	Group 1	0.89	0.65
		Group 2	0.66	0.59
	Non cleft side	Group 1	0.88	0.61
		Group 2	0.73	0.59

Natural smile	Cleft	Group 1	0.65	0.50
		Group 2	0.40	0.52
	Non cleft side	Group 1	0.72	0.55
		Group 2	0.42	0.52

Forced smile	Cleft	Group 1	0.53	0.42
		Group 2	0.30	0.47
	Non cleft side	Group 1	0.53	0.48
		Group 2	0.30	0.48

**Table 6:** Estimate of mean differences of lip thickness (ANOVA).

Effect	type	Estimate	Standard derivation	*P*	Inferior	Superior
Type	1-2	0.2407	0.02752	<.0001*	0.1587	0.3228
Type	1–3	0.3722	0.02752	<.0001*	0.2901	0.4543
Type	2-3	0.1315	0.02752	<.0001*	0.04941	0.2136
Group	1-2	0.2309	0.02247	<.0001*	0.1639	0.2979

*statistically significant (*P* < .05).

**Table 7:** Mean curve of the upper lip.

Side	Smile types	Examiner group	Mean score	Standard deviation
Cleft side	Natural	Group 1	1.84	0.50
		Group 2	1.68	0.41
	Forced	Group 1	1.83	0.59
		Group 2	1.60	0.43

Non cleft side	Natural	Group 1	1.82	0.54
		Group 2	1.66	0.40
	Forced	Group 1	1.85	0.59
		Group 2	1.68	0.52
